# Molecular-level investigation of CO_2_ absorption in deep eutectic solvents using molecular dynamics simulations and the COSMO-RS model

**DOI:** 10.1039/d6ra04027f

**Published:** 2026-07-03

**Authors:** Prateek Banerjee, Surya S Urs, Rima Biswas

**Affiliations:** a Process Simulation Research Group, School of Chemical Engineering, Vellore Institute of Technology Vellore Tamil Nadu 632014 India rima.biswas@vit.ac.in

## Abstract

This work proposes to thoroughly assess the thermodynamic and kinetic performance of deep eutectic solvents (DESs) based on choline chloride (ChCl) for CO_2_ immobilization in order to meet the urgent need for effective carbon capture technologies. Molecular dynamics (MD) simulations and the Conductor-like Screening Model for Real Solvents (COSMO-RS) were used to determine the solubility of CO_2_ in three different DESs: ChCl–phenol, ChCl–diethylene glycol (DEG), and ChCl–triethylene glycol (TEG) at temperatures between 303 K and 323 K. The hydrogen bond acceptor (HBA) to hydrogen bond donor (HBD) mole ratios of 1 : 3 and 1 : 4 were chosen. The computational findings show that the ChCl–TEG system at a 1 : 4 molar ratio had the maximum CO_2_ absorption capacity, with simulated solubilities of 0.0054, 0.0047, and 0.0043 at 303, 313, and 323 K, respectively. Henry's law constants predicted by the COSMO-RS model rise with temperature, indicating strong thermodynamic agreement with MD simulations. Three-dimensional spatial distribution functions define a site-specific solvation shell in which Cl^−^ anions and HBD preferentially coordinate equatorially around the central carbon atom of CO_2_, whereas [Ch]^+^ cations are localized along the axial termini near the oxygen atoms. Furthermore, CO_2_ diffusivity increases as temperature rises, demonstrating a common kinetic-thermodynamic trade-off. This computational study provides a definitive, molecular-level understanding of how the constituent cations, anions, and HBDs of DESs affect CO_2_ absorption.

## Abstract 2

In this work, molecular dynamics simulations and the COSMO-RS model were employed to investigate the CO_2_ absorption behavior of ChCl-based DESs, providing microscopic insights into the interactions governing gas capture.

## Graphical abstract

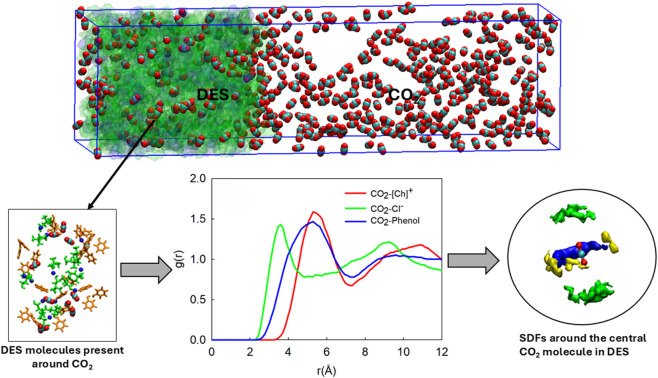

## 1. Introduction

Concerns about global warming have drawn interest from both industry and academia in carbon dioxide (CO_2_) capture and storage (CCS).^1,2^ The most popular CO_2_ capture technique in use currently is chemical absorption by aqueous solutions of alkanolamines, such as methyldiethanolamine (MDEA), diethanolamine (DEA), and monoethanolamine (MEA).^3,4^ However, this procedure has three significant disadvantages: (i) severe equipment corrosion; (ii) high energy costs for solvent regeneration; and (iii) significant solvent loss.^5,6^ Therefore, it is very desirable to look for functionally competitive and environmentally benign substitutes for alkanolamines from the perspectives of green chemistry and sustainability.

Ionic liquids (ILs) have garnered significant attention as substitute solvents for CO_2_ absorption in the past few years because of their distinct physicochemical characteristics, which include low vapor pressure, wide liquid range, and high thermal and chemical stability.^7–10^ The use of IL-based technology for CO_2_ separation in industrial processes is difficult due to issues including high viscosity, expensive synthesis and purification methods.^11,12^ In addition, there are rising worries about the toxicity of several conventional ILs. DESs were proposed as flexible substitutes to address the drawbacks of ILs.^13^ They are a novel type of ionic liquids that have a melting point significantly lower than any of their constituent parts.^14^ They are made by combining metal halides or hydrogen bond donors with a substituted quaternary ammonium salt. DESs have at least one hydrogen bond acceptor and one HBD counterpart.^15^ The selection of the nature and the ratio of the hydrogen bond counterparts can modify their properties. They frequently have a lot of fascinating properties in common with green solvents, such as biodegradability and low cost. A DES was first reported through the combination of urea and choline chloride.^16^ Additional analogous DESs were created and utilized in several domains, including liquid separations, metal electrodeposition, and as catalysts or solvents in bio-transformations.^17–19^ Due to their advantageous characteristics, DESs have been investigated in the field of gas capture through a number of experimental studies.^20–22^ Li *et al.*^23^ studied the effect of pressure and temperature on CO_2_ solubility at various urea and choline–chloride compositions. Leron *et al.*^20^ investigated the solubility of CO_2_ at different temperatures and pressures using a eutectic mixture of ChCl–ethylene glycol (in molar ratio 1 : 2), known as ethaline. The solubility values recorded were within the normal range as typically found in ILs. Superbases are useful for CO_2_ sequestration when added to ChCl-based DESs, as reported by Bhawna *et al.*^21^ The authors demonstrated that the inclusion of superbases enhanced the CO_2_ capture efficiency of ethaline and reline DESs. The CO_2_ capture using DES made of ChCl and natural lactic acid (1 : 2 mole ratio) was reported by Francisco *et al.*^24^

DES-based CO_2_ absorption studies have mostly measured CO_2_ solubility in DESs, covering only a small number of possibilities due to the numerous HBA–HBD combinations at various ratios.^25,26^ It is costly, time-consuming, and possibly impractical to use the experimental trial-and-error method to investigate more optimal DESs over a wide area. A reliable theoretical model to predict CO_2_ absorption capacity in DESs is essential.^27^ Equations of state techniques (such as PR-EOS, soft-SAFT, and PC-SAFT) and conventional thermodynamic models (such as NRTL and UNIQUAC) have been effectively applied to DES-containing systems as reliable predictors of gas solubility in recent years.^28–30^ However, these approaches require experimentally fitted molecule-specific and mixing parameters, limiting their applicability to novel systems. The COSMO-based thermodynamic models (COSMO-RS and COSMO-SAC) use molecular descriptors developed from quantum chemistry to accurately estimate CO_2_ solubility in IL solvents.^31,32^ In this regard, Liu *et al.*^33^ systematically tested and evaluated the predictive capacity of COSMO-RS by compiling a literature dataset that included 502 experimental points for CO_2_ solubilities and 132 data points for Henry's law constants in DESs. Pour *et al.*^34^ also used the COSMO-RS model to determine the Henry's law constants and solubility of acid gases (H_2_S/CO_2_) in quaternary ammonium salt-based DESs. Their findings show that these ammonium quaternary salt (AQS)-based DESs significantly enhance competitive gas separation efficiencies, highlighting the importance of COSMO-RS in designing high-performance solvent systems. However, these models significantly overestimate or underestimate gas solubility (CO_2_, CO, CH_4_, H_2_, and N_2_) in DESs.^35^ Molecular simulation techniques, such as Monte Carlo simulations and molecular dynamics simulations, are accurate in predicting the phase equilibrium or thermo-physical characteristics of DESs, including their gas solubility in solvents.^36,37^ The efficiency of choline chloride-based DESs on CO_2_ absorption using molecular dynamics (MD) simulations and density functional theory (DFT) was studied by Aparicio and co-workers.^38^ García *et al.*^39^ investigated the characteristics of ChCl-based DES at 318 K in the pure state as well as at the vacuum and gas interfaces using MD simulations. The authors additionally investigated the adsorption of pure CO_2_ and SO_2_ at the DES interface. They reported that these molecules achieved a parallel arrangement with the interface for optimal interactions with DES molecules. Altamash *et al.*^40^ investigated the effective CO_2_ capture by ChCl–phenylacetic acid DES at the interface using DFT and molecular dynamics simulations. Few studies have examined the solvation structure of gases in bulk DES, while most focus on the gas capture mechanism at the interface. Malik *et al.*^41^ investigated the solvation structure around SO_2_ and CO_2_ in ChCl-based DESs, namely, ethaline and reline, using *ab initio* MD simulations. Alizadeh *et al.*^42^ used *ab initio* MD to study the absorption mechanism of CO_2_ in ethaline at different pressure levels.

Significant CO_2_ absorption capacities are demonstrated by DESs of choline chloride and alcohols.^43^ However, there is limited research on the solvation structure of CO_2_ in DESs of choline chloride and phenol or alcohols. In the present study, we performed the MD simulations to understand the CO_2_ absorption mechanism in three different types of ChCl-based DESs, namely, ChCl–phenol, ChCl–DEG, and ChCl–TEG DESs at different temperatures and a pressure of 1 atm. DESs with 1 : 3 and 1 : 4 molar ratios were chosen for their stable hydrogen-bonding networks and favorable physicochemical properties for CO_2_ absorption, as reported in prior literature,^43^ where similar DES systems and molar ratios were used to experimentally investigate the CO_2_ solubility at various temperatures. As a result, these molar ratios were used in our current study to enable valid comparisons with available experimental data and to further evaluate molecular-level interactions using MD simulations. Furthermore, changing the HBD content has a major impact on the viscosity, free volume, and intermolecular interactions of DES systems, which directly affect the CO_2_ capture. Our aim is to demonstrate how DES's HBD affects CO_2_ sorption and diffusion at the microscopic level. The primary goals of the current study are to examine how temperature and HBD variation affect CO_2_ solubility in DESs. The interfacial behavior and transport dynamics of CO_2_ in DESs under ambient conditions were investigated using an independent MD simulation. Finally, the Henry's law constant of CO_2_ at different temperatures was predicted using the COSMO-RS model.

## 2. Computational methods

### 2.1. MD simulations

**Fig. 1:**
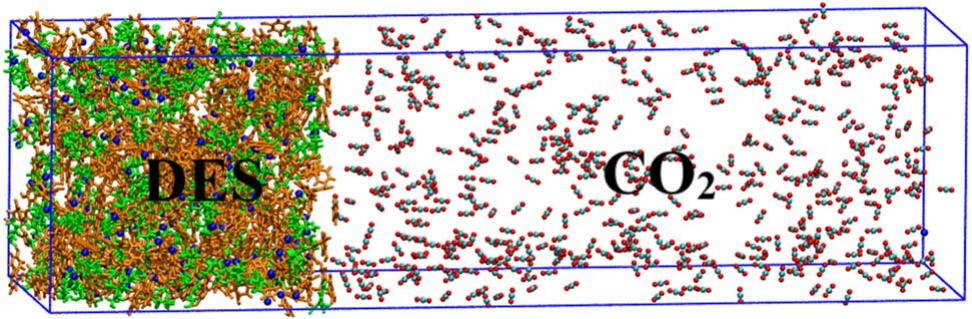
The initial configuration of the DES1–CO_2_ system for CO_2_ solubility calculations.

**Fig. 2:**
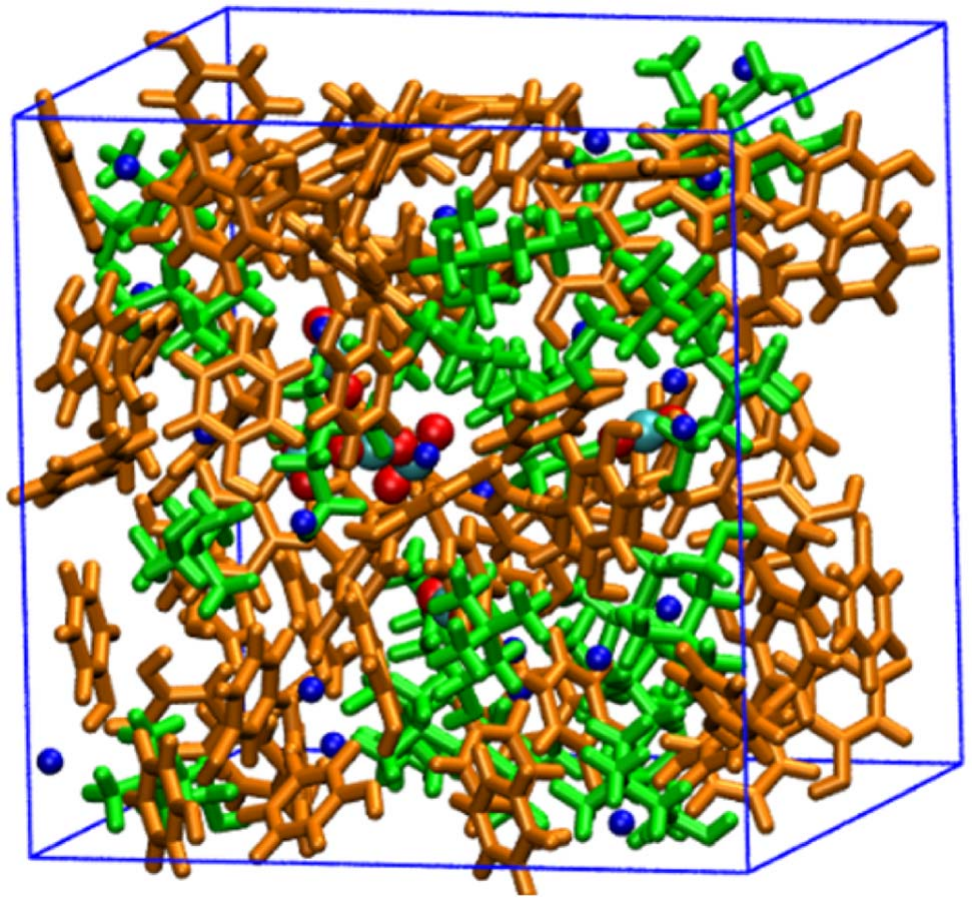
The initial snapshot of the DES1–CO_2_ system for the diffusivity calculations.

All the MD simulations were performed using NAMD software (version 2.14).^44^ Phenol, DEG, and TEG were employed as hydrogen bond donors, and ChCl as a hydrogen bond acceptor in this study. GaussView 5.0 constructs the initial structure of molecules, which are subsequently optimized using B3LYP and 6-31G* basis sets in Gaussian09 software.^45^ DES1 and DES2 indicate the 1 : 3 and 1 : 4 molar ratios of ChCl : phenol. DES3 and DES4 represent the 1 : 3 and 1 : 4 molar ratios of ChCl : DEG; and DES5 and DES6 represent the 1 : 3 and 1 : 4 ratios of ChCl : TEG. Simulations were carried out at different temperatures and at a pressure of 1 atm. The solvation box was created using PACKMOL^46^ software with a box diameter of 58 Å × 58 Å × 180 Å, as seen in Fig. 1. The simulation box is filled with bulk CO_2_ molecules in one half and bulk DES molecules in the other half to create the two-phase system. To determine the solubility of CO_2_ in DES, we have considered 500 molecules in the bulk CO_2_ phase and 200 molecules of ChCl and 600 molecules of HBD in the bulk DES phase for 1 : 3 molar ratios, whereas 800 molecules of HBD were used for 1 : 4 molar ratios. Table 1 summarizes the number of DES and CO_2_ molecules as well as box dimensions. The CO_2_ force field parameters were obtained from Potoff and Siepmann,^47^ while the parameters for ChCl-based DESs were extracted from Doherty and Acevedo.^48^ The Langevin piston and thermostat were used for controlling the pressure and temperature, respectively. To determine the long-range electrostatic interactions, the Ewald summation method was employed. The equations of motion were integrated using the Verlet algorithm with a time step of 1 fs. The most recent 10 ns trajectory was selected for investigation after the simulation ran for 50 ns in the NVT ensemble. When CO_2_ and DES were in equilibrium, the quantity of CO_2_ molecules in DES was used to compute solubility at ambient conditions. To investigate the molecular-level interactions between DES and CO_2_, we have considered 24 molecules of ChCl and 72 molecules of HBDs for 1 : 3 molar ratios and 96 molecules of HBDs for 1 : 4 molar ratios, as mentioned in Fig. 2. The initial DES systems were energy minimized for 10 000 steps to balance the potential energy distribution of molecules. The cooling procedure at 1 atm started at 70 K higher than the CO_2_ capture operating temperature and gradually decreased by 10 K intervals until it reached the CO_2_ capture operating temperature. The systems were equilibrated in an NPT ensemble at 1 atm for 10 ns. After NPT equilibration at the CO_2_ capture temperature, the final frame of the DES was exposed to CO_2_ molecules to simulate absorption in the DES. The absorption processes were carried out in the canonical ensemble (NVT) for 50 ns, resulting in uniform distribution of CO_2_ molecules. The distribution of CO_2_ was analyzed for the last 10 ns. The densities are calculated once the NPT ensemble reaches equilibrium (Table 2). The results closely correspond to the experimental data.^43^ The MD simulations for the bulk system only show the system at equilibrium. The images were taken using the VMD program.^49^ The TRAVIS software package^50^ was used to calculate the spatial distribution function (SDF), which shows the spatial distribution and structural packing of molecules.

**Table 1:** Numbers of DES and CO_2_ with box dimensions of DES–CO_2_ systems

Systems	No. of DESs	No. of CO_2_	Box size *x* × *y* × *z* (Å^3^)
DES1	ChCl (200), phenol (600)	500	58.23 Å × 58.23 Å × 180.08 Å
DES2	ChCl (200), phenol (800)	500	58.14 Å × 58.14 Å × 180.25 Å
DES3	ChCl (200), DEG (600)	500	58.17 Å × 58.17 Å × 180.42 Å
DES4	ChCl (200), DEG (800)	500	58.33 Å × 58.33 Å × 180.06 Å
DES5	ChCl (200), TEG (600)	500	58.07 Å × 58.07 Å × 180.13 Å
DES6	ChCl (200), TEG (800)	500	58.26 Å × 58.26 Å × 180.28 Å

**Table 2:** Comparing the experimental and simulated properties of DESs at 303 K and 1 atm

Systems	*ρ*/(g cm^−3^) MD simulation	*ρ*/(g cm^−3^) experiment^43^
DES1	1.093 ± 0.037	1.087
DES2	1.088 ± 0.011	1.085
DES3	1.119 ± 0.098	1.117
DES4	1.115 ± 0.023	1.113
DES5	1.123 ± 0.067	1.126
DES6	1.112 ± 0.055	1.114

The CO_2_ solubility in DESs is determined by the following equation,^51^1
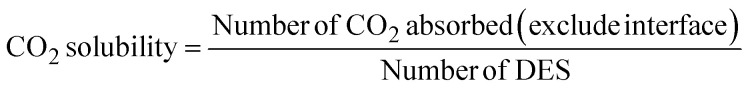


The radial distribution functions and coordination numbers are calculated using eqn (2) and (3),^52,53^2
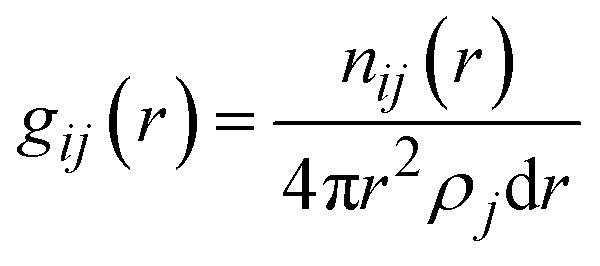
3
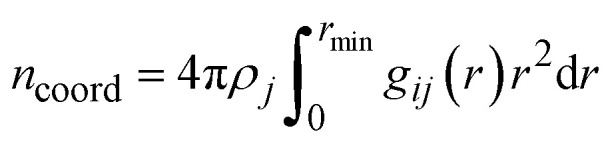
where *n*_*ij*_(*r*) is the ensemble average of all the *j*th-type particles in the small volume element 4π*r*^2^d*r* at a distance of *r* from the central *i*th-type particle. *ρ*_*j*_ is the bulk number density of *j*th-type particles. *r*_min_ represents the first minimum positions in RDFs.

The self-diffusion coefficient of CO_2_ in solvents was calculated by the Einstein equation.^54^4
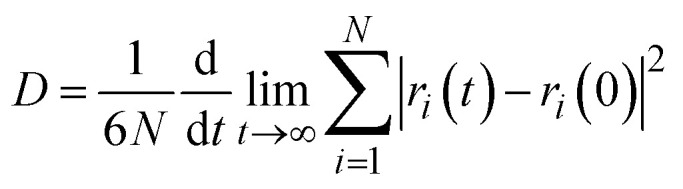


The positions of atom *i* at time 0 and *t* are given as *r*_*i*_(0) and *r*_*i*_(*t*), respectively. The slope was calculated from the mean square diffusivity (MSD) to get the CO_2_ self-diffusion coefficient. The diffusivity of CO_2_ was determined using the last 10 ns of the trajectory.

### 2.2. COSMO-RS model

The thermodynamic properties of a fluid mixture were calculated using the COSMO-RS model. Klamt *et al.*^55,56^ published the model first. The Amsterdam Modeling Suite (AMS) version AMS2024.106 was used for COSMO-RS computations.^57^ The molecular ADF program in AMS was used for the initial QC COSMO calculations of σ-profiles, whereas the AMS COSMO-RS program was used for the subsequent thermodynamic calculations. The AMS software includes its own database of precalculated σ-profiles. The geometry was improved at the QC level with DFT BP86/pVTZ in the ADF package of AMS.^58^ The ADF program's default preset “COSMO-RS Compound” executes COSMO-RS parametrization of molecules, including the later stage.^58^ The second stage of “COSMO-RS Compound” uses the COSMO solvation model and Delley cavity construction to calculate QC and build a σ-profile. The σ-profile for CO_2_ was obtained from the “ADF-CRS2018” database, which was distributed alongside the AMS software.^57^ The COSMO file shows the screening charge density of the molecule. The screen charge densities dictate how the molecule surfaces interact with one another. The molecular surface is separated into segments based on their average screening charge. The screening charges are shown as probability distributions or as a histogram.^55^ A sigma profile is then created by combining these screening charges. The activity coefficient can be calculated from these sigma profiles based on a statistical thermodynamic framework.^55^

The activity coefficient is calculated using eqn (5),5
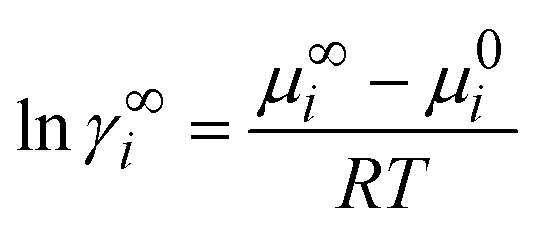
where *γ*^∞^_*i*_ is the activity coefficient for the compound *i* at infinite dilution. *µ*^0^_*i*_ and *µ*^∞^_*i*_ represent the pseudo-chemical potential of *i* in the pure liquid state and the chemical potential of *i* at infinite dilution state. Henry's law constants define the solubility of CO_2_ in the solvent. Henry's law constants^59^ can be determined using eqn (6),6
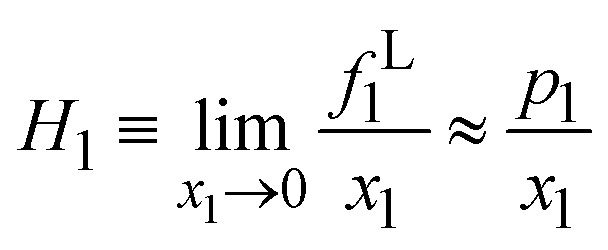
Here *H*_1_ represents Henry's law constant, *x*_1_ is the mole fraction of CO_2_ dissolved in the solvent, the fugacity of the vapor in the solvent is indicated by *f*^L^_1_, and the pressure of CO_2_ is represented by *p*_1_.

## 3. Results and discussions

Three kinds of DES systems with two different molar ratios, *i.e.*, 1 : 3 and 1 : 4, were considered for this study. Initially, we focused on the molecular structure of DES molecules and CO_2_ at the interface, followed by an examination of density profiles, CO_2_ solubilities, radial distribution functions (RDFs), and spatial distribution functions (SDFs) in ILs. This is the first study to use MD simulations and COSMO-RS calculations to analyze the impact of HBD type (phenol, DEG, and TEG) and HBA : HBD molar ratio (1 : 3 and 1 : 4) on CO_2_ absorption processes in ChCl-based DESs. The study indicates strong correlations between molecule–scale interactions, interfacial behavior, transport properties, and thermodynamic performance, giving molecular design principles for developing high-performance DESs for carbon capture.

### 3.1. Molecular structures at the DES–CO_2_ interface

**Fig. 3:**
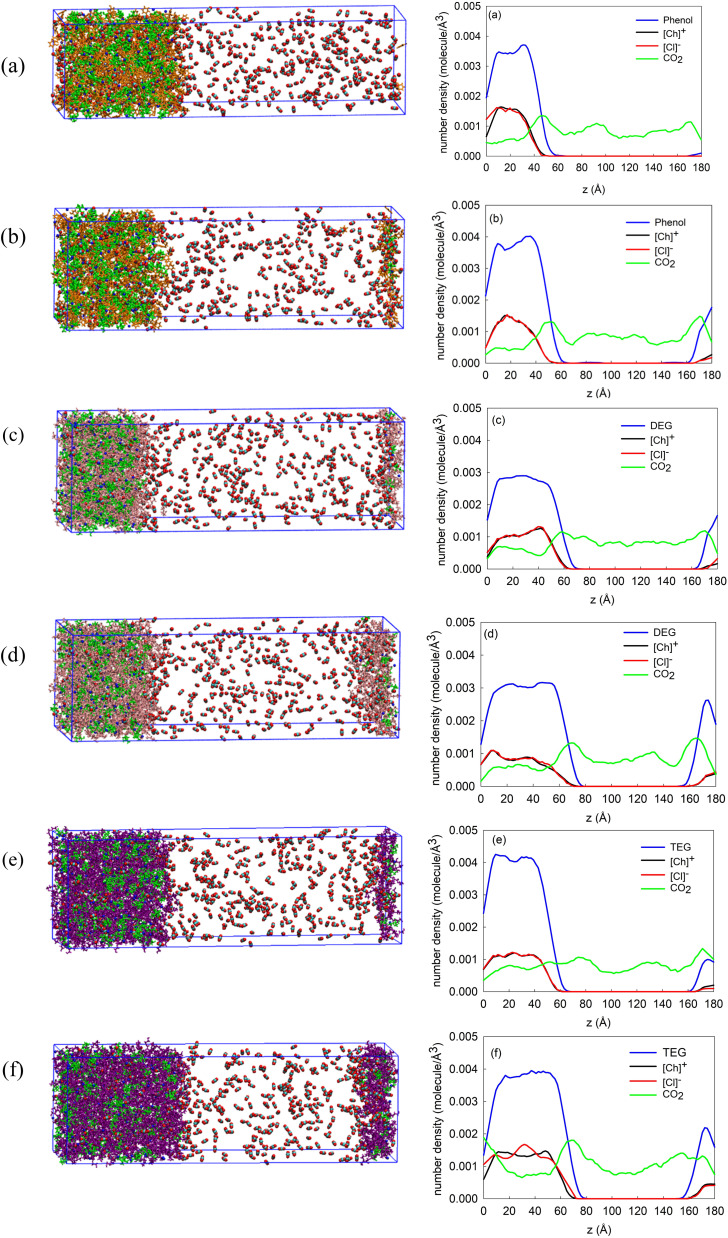
Final images of (a) DES1, (b) DES2, (c) DES3, (d) DES4, (e) DES5, and (f) DES6 at 303 K and 1 atm. Each system's number density profile for CO_2_ and DESs is listed on the right.

When a gas molecule approaches a liquid from the vapor phase, it first comes into contact with its interface. The gas's interaction with the species/functional group at the interface determines whether it is rejected from the surface or accommodated into the liquid. It is crucial to understand the composition, structure, and molecular orientation at the interface. We examine the number density profile along the interfacial normal direction based on their *z*-positions. Fig. 3 shows the final snapshots and the density profiles for DES1–CO_2_, DES2–CO_2_, DES3–CO_2_, DES4–CO_2_, DES5–CO_2_, and DES6–CO_2_ binary mixed systems. The DES6 system has a maximum average CO_2_ density of 1.08 × 10^−3^ Å^−3^ in the liquid phase, while DES1, DES2, DES3, DES4, and DES5 have values of 6.62 × 10^−4^ Å^−3^, 6.17 × 10^−4^ Å^−3^, 6.33 × 10^−4^ Å^−3^, 5.88 × 10^−4^ Å^−3^ and 6.79 × 10^−4^ Å^−3^ at 303 K and 1 atm, respectively. CO_2_ molecules are preferentially accumulated at the gas–liquid interface, as indicated by the greatest density of CO_2_ at the interface. An experiment by Roscioli and Nesbitt^60^ revealed similar behavior. According to Roscioli and Nesbitt,^60^ interfacial CO_2_ accumulation is caused by strong CO_2_-IL interaction, while CO_2_ sorption in IL is controlled by the free volume of IL. The solubility of CO_2_ in DESs at particular temperatures and pressures is also connected with the density profile.

Table 3 shows the findings of MD simulations that were performed to evaluate the solubility of CO_2_ in a number of DESs at ambient conditions. The quantity of CO_2_ absorbed in DESs was calculated without taking into account the amount of CO_2_ at the interface. We have determined the solubility at 1 atm and various temperatures (*i.e.*, 303 K, 313 K, and 323 K). According to the computed solubility, at various temperatures, the CO_2_ solubility in the DES6 system (0.0054, 0.0047, and 0.0043) is greater than that in other DES systems. As seen in Table 3, the solubility results agreed with the experimental data. The efficiency of DES for CO_2_ capture at different temperatures was examined, and it was discovered that the solubility reduced as the temperature increased. This solubility phenomenon could suggest a physical dissolution procedure for CO_2_ capture. When the HBD changes from [DEG] to [phenol] to [TEG], the solvation of CO_2_ gets more favorable. Furthermore, out of all the DESs, the ones made of ChCl and triethylene glycol with a mole ratio of 1 : 4 showed the greatest ability to dissolve CO_2_.

**Table 3:** Simulated CO_2_ solubility in different DESs compared with experiments^43^ at 1 atm

Systems	*T* = 303 K		*T* = 313 K		*T* = 323 K	
	*S*_MD_	*S*_Exp_	*S*_MD_	*S*_Exp_	*S*_MD_	*S*_Exp_
DES1	0.0041	0.0035	0.0037	0.0032	0.0033	0.0029
DES2	0.0039	0.0035	0.0035	0.0032	0.0032	0.0027
DES3	0.0036	0.0037	0.0033	0.0030	0.0027	0.0025
DES4	0.0037	0.0034	0.0035	0.0030	0.0028	0.0023
DES5	0.0051	0.0047	0.0045	0.0038	0.0041	0.0034
DES6	0.0054	0.0049	0.0047	0.0042	0.0043	0.0033

### 3.2. Structural properties of CO_2_ in DESs

**Fig. 4:**
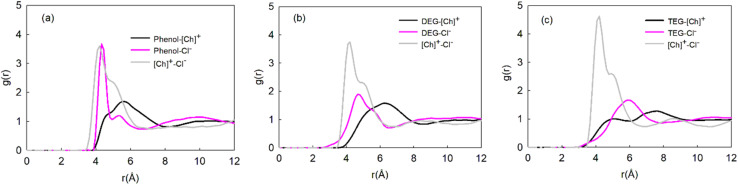
Radial distribution functions between the different species present in pure (a) ChCl–phenol (1 : 3), (b) ChCl–DEG (1 : 3), and (c) ChCl–TEG (1 : 3).

**Fig. 5:**
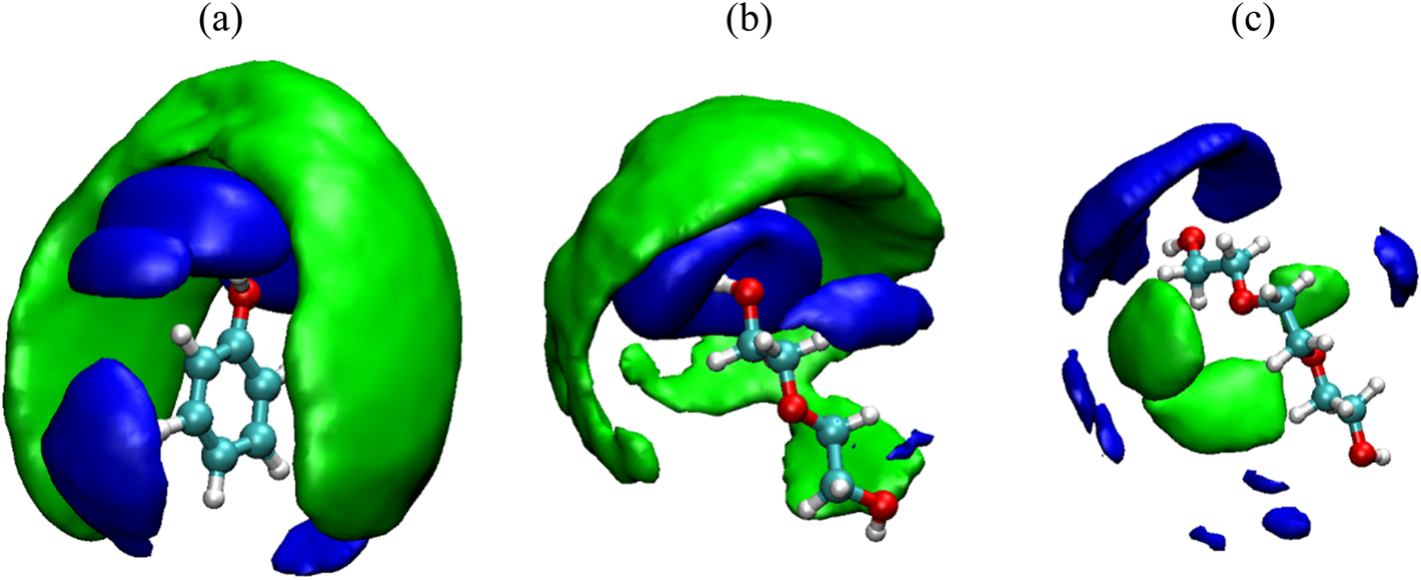
Spatial distribution functions for (a) phenol, (b) DEG, and (c) TEG in ChCl-based deep eutectic solvents. Green color denotes the isosurface of the choline cation (isovalue is 2.62), and blue color depicts the isosurface of the chloride anion (isovalue is 1.95).

To better understand the solubility trend in DESs, we estimated the radial distribution function of cations, anions, and HBDs. Fig. 4 shows the center-of-mass radial distribution functions for pure DES systems at 1 : 3 ratios. The higher peaks with lower *r* imply the higher interaction; therefore, ChCl–TEG (1 : 3) DES has the weakest interaction between HBD and cation or anion, allowing CO_2_ to easily fit into the interstitial cavities inside the DES. The RDFs further support the spatial distribution functions of [Ch]^+^ and Cl^−^ ions around the HBDs. We have calculated the spatial distribution functions using the 5000 configurations from the last 10 ns trajectory to understand the structural arrangements of cations and anions near the HBD molecules (Fig. 5). The distribution of [Ch]^+^ and Cl^−^ around the active side of the TEG was found to be smaller than that of phenol and DEG, according to the SDF calculation. This shows that the TEG has larger cavities and less interactions with cations and anions. Therefore, the simulated and experimental CO_2_ solubility trends appear to be correlated with both radial and spatial distribution functions, showing that larger cavities result in higher CO_2_ solubility.

**Fig. 6:**
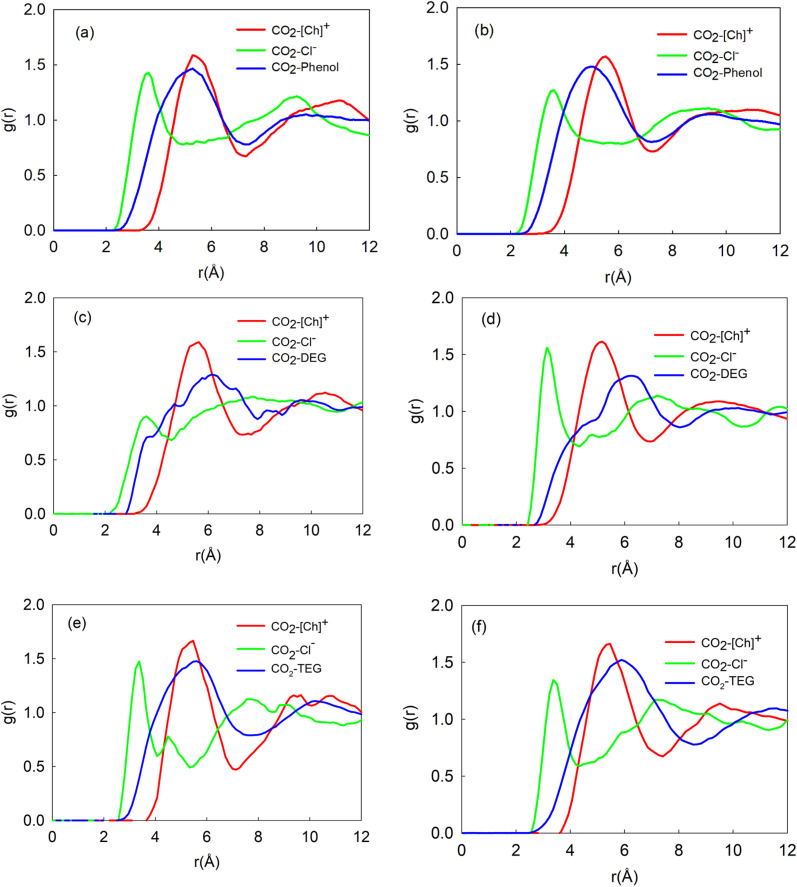
Center of mass radial distribution functions of CO_2_ with cation ([Ch]^+^), anion (Cl^−^), and HBDs (phenol, DEG, and TEG) in the (a) DES1, (b) DES2, (c) DES3, (d) DES4, (e) DES5, and (f) DES6 systems.

To analyze the structural properties of CO_2_ in various DESs, we computed the center of mass RDFs for CO_2_ with anion, cation, and HBD using the last 10 ns simulation trajectory. The RDF for all DES systems is displayed in Fig. 6. In every system, we discovered that the Cl^−^ anion is closest to the CO_2_ molecules. Next, come to the [Ch]^+^ cations and HBD, which lie around 5.4 Å and 5.6 Å. The Cl^−^ anion is very near the CO_2_ molecules for DES5 and DES6 systems, with a peak at *r* = 3.38 Å and *r* = 3.35 Å. In other words, the high peak value and small *r* of TEG for DES5 (1.48, 5.61 Å) and DES6 (1.53 and 5.68 Å) systems indicate that TEG molecules are highly interacted with CO_2_ molecules. The higher peak height of CO_2_ with [Ch]^+^ cation for DES5 (1.66, 5.43 Å) and DES6 (1.68, 5.42 Å) systems indicates stronger interactions between [Ch]^+^ and CO_2_ molecules. The CO_2_–DES RDFs were integrated until the first minimum was reached in order to determine the coordination number of DES molecules in the first solvent shell of CO_2_. The outcome is listed in Table 4. Comparing the RDF peaks reveals how the anion, cation, and HBD species are arranged in the first solvation shell of the CO_2_ molecule in DESs. The average coordination numbers of phenol, DEG, and TEG molecules for 1 : 3 molar ratio systems (DES1, DES3, and DES5) at 303 K and 1 atm are 12.15, 9.69, and 13.15. In other words, the average coordination numbers of these HBD molecules at the same temperature and pressure for 1 : 4 molar ratio systems (DES2, DES4, and DES6) are 11.43, 10.49, and 14.73. This result indicates that phenol molecules strongly interact with CO_2_ molecules at a 1 : 3 molar ratio system (DES1), whereas DEG and TEG molecules strongly interact with CO_2_ at 1 : 4 molar ratio systems (DES4 and DES6). Similar conditions we observed for Cl^−^ and [Ch]^+^, also, where the higher coordination number for anions (1.47) and cations (4.32) in a 1 : 3 molar ratio of ChCl–phenol systems (DES1) indicates strong interaction between CO_2_ and cations/anions. On the other hand, the coordination number for [Ch]^+^ and Cl^−^ was found to be higher for 1 : 4 molar ratio of ChCl–DEG (DES4) and ChCl–TEG systems (DES6) as compared to 1 : 3 molar ratio systems (DES3 and DES5). This result suggests that the molar ratio of HBA to HBD also affects the CO_2_ absorption process.

**Table 4:** Average coordination numbers of the first CO_2_ solvation shells in various DES systems

Systems	Cl^−^ around CO_2_	[Ch]^+^ around CO_2_	HBD around CO_2_
DES1	1.47 ± 0.08	4.32 ± 0.01	12.15 ± 0.01
DES2	1.11 ± 0.05	3.97 ± 0.07	11.43 ± 0.06
DES3	0.50 ± 0.06	3.51 ± 0.07	9.69 ± 0.05
DES4	0.85 ± 0.02	3.82 ± 0.03	10.49 ± 1.03
DES5	1.53 ± 0.08	4.56 ± 0.05	13.15 ± 0.08
DES6	1.64 ± 0.01	5.18 ± 0.09	14.73 ± 0.08

**Fig. 7:**
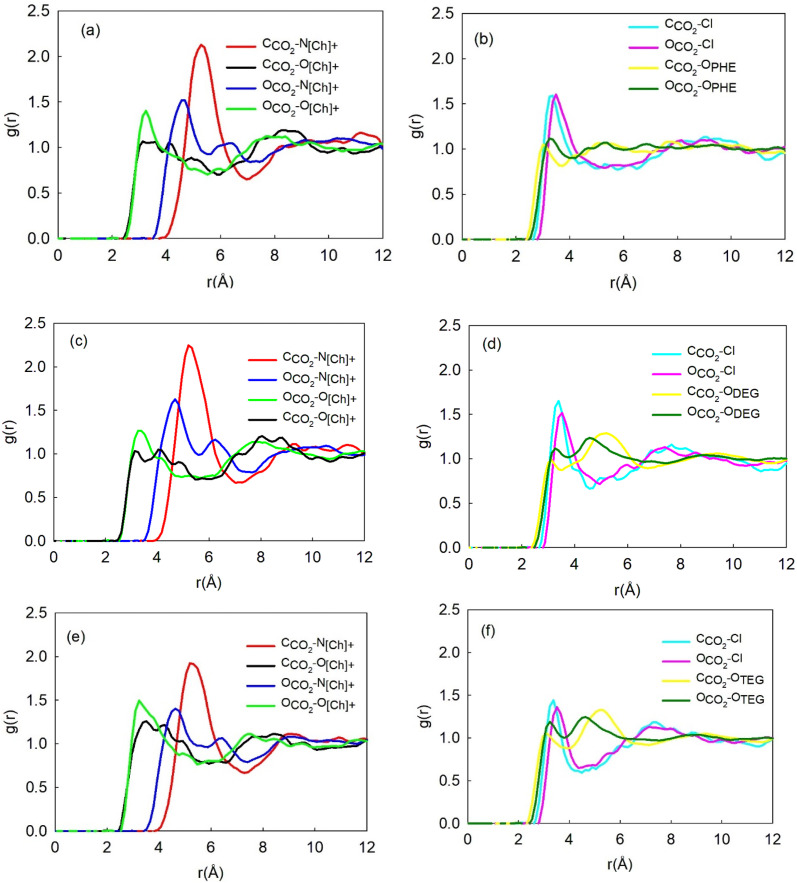
Radial distribution functions for (a) C_CO_2__–N_[Ch]^+^_, C_CO_2__–O_[Ch]^+^_, O_CO_2__–N_[Ch]^+^_, and O_CO_2__–O_[Ch]^+^_ and (b) C_CO_2__–Cl, O_CO_2__–Cl, C_CO_2__–O_PHE_, and O_CO_2__–O_PHE_ in DES1 systems; (c) C_CO_2__–N_[Ch]^+^_, C_CO_2__–O_[Ch]^+^_, O_CO_2__–N_[Ch]^+^_, and O_CO_2__–O_[Ch]^+^_ and (d) C_CO_2__–Cl, O_CO_2__–Cl, C_CO_2__–O_DEG_, and O_CO_2__–O_DEG_ in DES3 systems; (e) C_CO_2__–N_[Ch]^+^_, C_CO_2__–O_[Ch]^+^_, O_CO_2__–N_[Ch]^+^_, and O_CO_2__–O_[Ch]^+^_ and (f) C_CO_2__–Cl, O_CO_2__–Cl, C_CO_2__–O_TEG_, and O_CO_2__–O_TEG_ in DES5 systems.

To comprehend how DES molecules are arranged around CO_2_, we calculated atomic RDFs for the interactions of DESs with CO_2_. In atomic RDF calculations, we have considered three DES systems, *i.e.*, DES1, DES3, and DES5, respectively. Fig. 7 displays the atomic RDFs for the CO_2_ interaction with the [Ch]^+^, Cl^−^, and HBD. The atomic RDFs for the interaction between [Ch]^+^ and the CO_2_ molecule in the DES1 system are displayed in Fig. 7a. Comparing the height of the primary peaks, we discovered that the nitrogen atom of [Ch]^+^ (N_[Ch]^+^_) is more closely related to the carbon atom of CO_2_ (C_CO_2__). However, compared to the carbon atom of CO_2_ (C_CO_2__), the oxygen atom of [Ch]^+^ (O_[Ch]^+^_) is closer to the oxygen atom of CO_2_ (O_CO_2__). The closer association of O_CO_2__ with chlorine anion is displayed in Fig. 7b, whose peaks are located at 3.48 Å. The oxygen atom of phenol (O_PHE_) is more closely related to O_CO_2__ than the C_CO_2__. The atomic RDFs for the major interactions between the CO_2_ molecule and DES3 solvents are displayed in Fig. 7c and d. We observed a closer association of N_[Ch]^+^_ with C_CO_2__, displaying the higher peak (2.26) at 5.18 Å compared to the RDF peak of N_[Ch]^+^_–O_CO_2__ (1.62) at 4.68 Å. The O_[Ch]^+^_ is found to be more closely related to O_CO_2__ than C_CO_2__, with the RDF peak at 3.22 Å. The carbon atom of CO_2,_*i.e.*, C_CO_2__ is closely interacted with Cl^−^, showing the sharp RDF peak at 3.48 Å, whereas the oxygen atom of DEG (O_DEG_) displays a stronger correlation with the oxygen atom of CO_2,_ with the RDF peak at 3.26 Å. Fig. 7d and f show the atomic RDFs for the DES5 solvent. We observed a stronger correlation between N_[Ch]^+^_ and C_CO_2__, with a sharp RDF peak at 5.22 Å compared to the N_[Ch]^+^_–O_CO_2__ RDF peak at 4.64 Å. In the same way, we discovered that Cl^−^ is closer to C_CO_2__ than O_CO_2__.

**Fig. 8:**
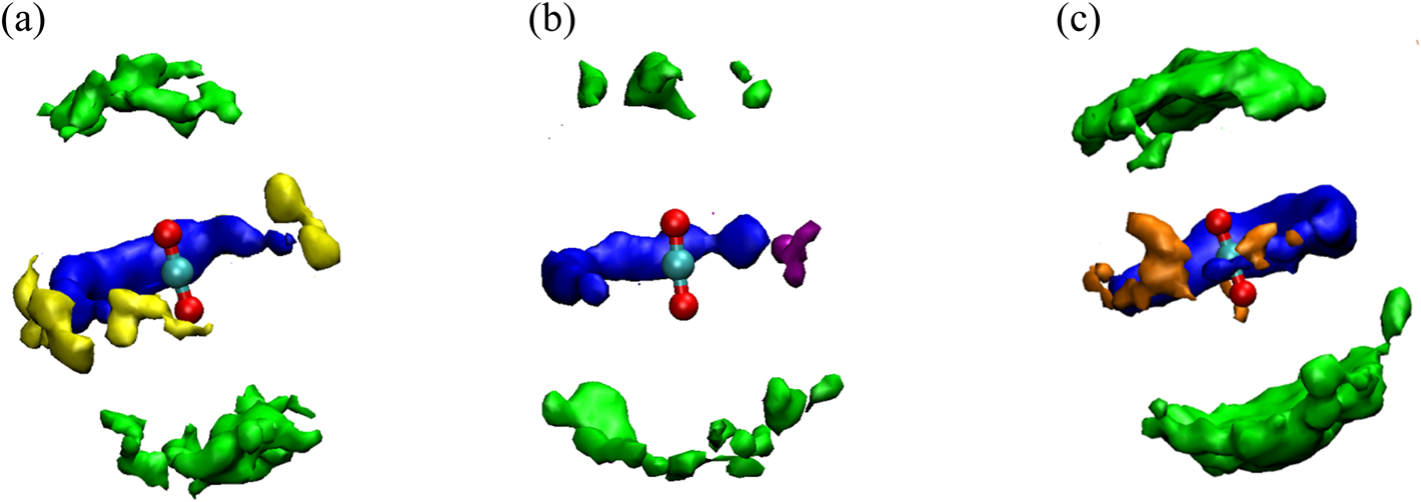
SDFs around the central CO_2_ molecule in (a) ChCl–phenol, (b) ChCl–DEG, and (d) ChCl–TEG at 303 K and 1 bar. Here, green, blue, yellow, purple, and orange isosurfaces represent [Ch]^+^ (isovalue is 3.10), Cl^−^ (isovalue is 2.75), phenol (isovalue is 9.48), DEG (isovalue is 7.03), and TEG molecules (isovalue is 5.85), respectively.

The preferred location of the DES molecules around the CO_2_ can be further understood using spatial distribution functions (SDFs). The SDFs for each DES were generated and examined using the TRAVIS software.^50^ In Fig. 8, the average density of anions, cations, and HBDs surrounding a reference CO_2_ molecule at 303 K and 1 atm is provided by the spatial distribution functions (SDFs). It is evident that the carbon atom of CO_2_ is encased in a circular shell made of an average anion and HBD species. The presence of the cation iso-surface on both sides of the CO_2_ oxygen atom suggests that the cation interacts with the CO_2_ oxygen atom in the first solvation shell. The isosurface of cations and anions is represented by the green and blue colors. The isosurface of phenol, DEG, and TEG molecules around the CO_2_ molecule is shown by the yellow, purple, and orange colors (Fig. 8a–c). TEG had a strong interaction with CO_2_ molecules parallel to the O–C–O line (axis), according to the SDFs. The coordination numbers and RDF results accord well with the SDF results.

### 3.3. Interaction energy

MD simulations were used to determine the CO_2_ interaction energy in various DESs, as indicated in Table 5. The interaction energy of CO_2_ with anions, cations, and HBDs is determined using Coulomb interactions and Lennard-Jones potentials. In DES1 and DES2 systems at 303.15 K and 1 atm, the interaction energies for CO_2_–cation, CO_2_–anion, and CO_2_–phenol are −14.34 kcal mol^−1^, −2.76 kcal mol^−1^, −11.12 kcal mol^−1^, −8.55 kcal mol^−1^, −1.45 kcal mol^−1^, and −9.18 kcal mol^−1^. The interaction energy of CO_2_–phenol is significantly higher than that of CO_2_–[Ch]^+^ and CO_2_–Cl^−^ due to the favorable electrostatic interaction between CO_2_ and phenol. The interaction energy between CO_2_ and DES3 and DES4 molecules was calculated, and it was observed that the interaction energy for CO_2_–cation, CO_2_–anion, and CO_2_–DEG was found to be higher for 1 : 4 molar ratios of ChCL–DEG systems (DES4) compared to 1 : 3 molar ratios of the system (DES3). The interaction energies for CO_2_–cation (−16.73 kcal mol^−1^), CO_2_–anion (−3.37 kcal mol^−1^), and CO_2_–TEG (−22.23 kcal mol^−1^) in DES6 systems were found to be higher than those of other DES systems. We found that the interaction energy between CO_2_ and HBD is higher than that of cations and anions in all systems. The interaction energies agree with the solubility results.

**Table 5:** The interaction energies of CO_2_ with cation, anion, and HBD in different DESs

Systems	Species	*E*_int_ (kcal mol^−1^)
DES1	[Ch]^+^–CO_2_	−10.34 ± 0.7
	Cl^−^–CO_2_	−2.76 ± 0.1
	Phenol–CO_2_	−15.12 ± 0.5
DES2	[Ch]^+^–CO_2_	−8.55 ± 0.1
	Cl^−^–CO_2_	−1.45 ± 0.1
	Phenol–CO_2_	−9.18 ± 0.7
DES3	[Ch]^+^–CO_2_	−7.29 ± 0.6
	Cl^−^–CO_2_	−0.43 ± 0.1
	DEG–CO_2_	−8.03 ± 0.5
DES4	[Ch]^+^–CO_2_	−8.21 ± 0.2
	Cl^−^–CO_2_	−1.02 ± 0.1
	DEG–CO_2_	−10.56 ± 0.8
DES5	[Ch]^+^–CO_2_	−13.56 ± 0.4
	Cl^−^–CO_2_	−2.84 ± 0.1
	TEG–CO_2_	−17.04 ± 0.3
DES6	[Ch]^+^–CO_2_	−16.73 ± 0.6
	Cl^−^–CO_2_	−3.37 ± 0.1
	TEG–CO_2_	−22.23 ± 0.8

Table 6 reports the average hydrogen bonds (HBs), which provide additional information on the nature of the interactions between the CO_2_ and DESs. Strong interactions between CO_2_ molecules and HBDs/cations result in the creation of HBs between them. Table 6 displays the average HBs over the last 10 ns trajectories. For DES1, DES2, DES3, DES4, DES5, and DES6, the average HBs between CO_2_ and cation are 2.5, 1.2, 1.1, 2.3, 3.5, and 4.4, respectively. The average HBs for CO_2_–cation is higher for the DES6 system as compared to other systems. Similarly, the average HBs for between CO_2_ and HBD is found to be higher for DES6 systems (7.5), showing strong interactions between CO_2_ and the TEG molecule at the 1 : 4 molar ratio of DES composed of ChCL and TEG.

**Table 6:** The average hydrogen bond numbers for CO_2_ with cation and HBD in the different DESs

Systems	CO_2_–cation	CO_2_–HBD
DES1	2.5 ± 0.02	3.8 ± 0.06
DES2	1.2 ± 0.01	2.2 ± 0.04
DES3	1.1 ± 0.03	1.7 ± 0.03
DES4	2.3 ± 0.05	3.4 ± 0.05
DES5	3.5 ± 0.03	5.1 ± 0.05
DES6	4.4 ± 0.03	7.5 ± 0.04

### 3.4. Diffusivity of CO_2_ in DESs

**Fig. 9:**
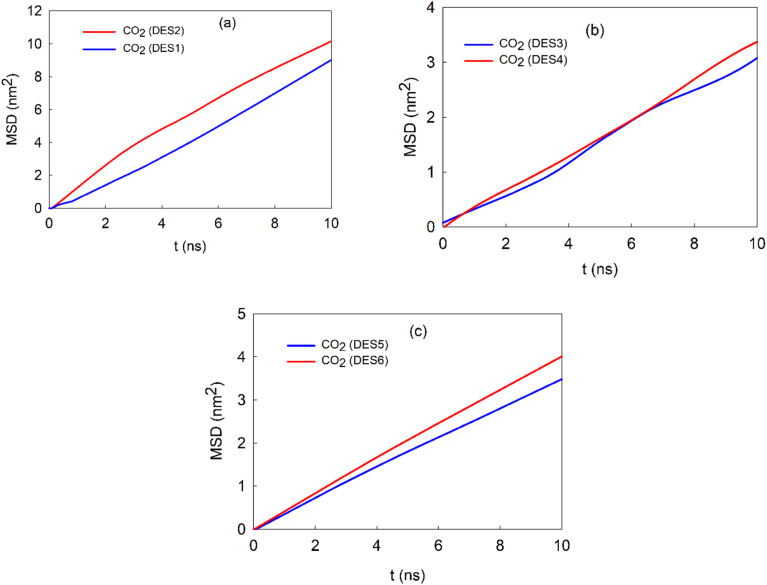
Mean square displacement for CO_2_ in (a) DES1 and DES2, (b) DES3 and DES4, and (c) DES5 and DES6 systems at 303.15 K and 1 atm.

**Fig. 10:**
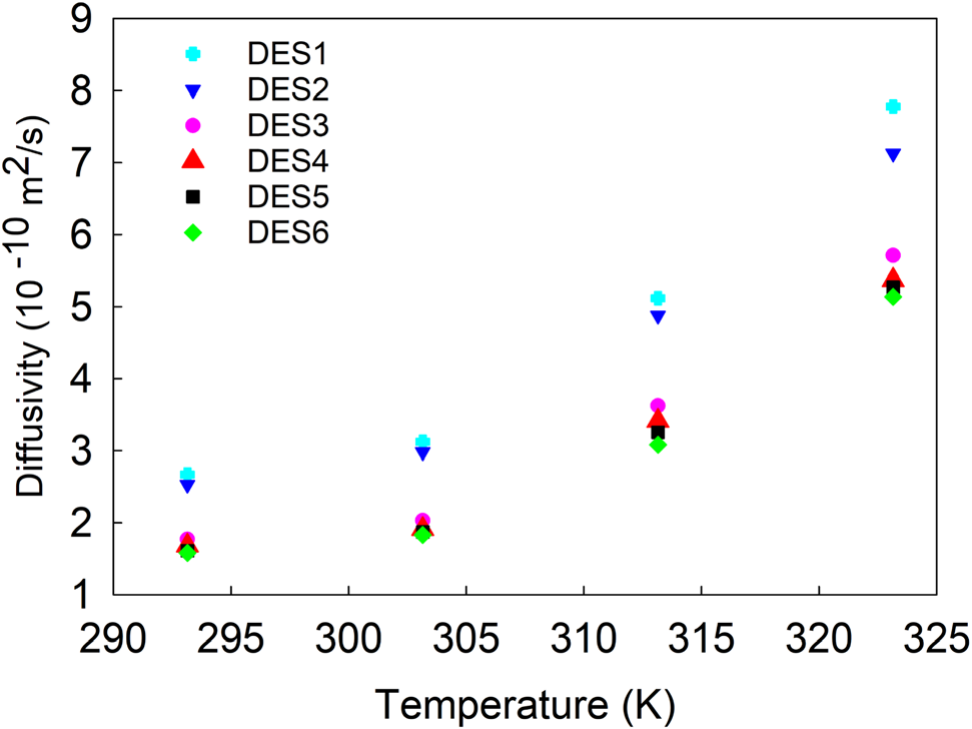
Diffusion coefficients of CO_2_ in different DESs.

The mean square displacement (MSD) model was used to determine the diffusion coefficients of CO_2_. Fig. 9 shows the MSD profiles of CO_2_ in various DES systems at 303.15 K. The diffusion coefficients of CO_2_ in six distinct DESs have been calculated in relation to temperature as illustrated in Fig. 10. The CO_2_ diffusivities in DES1, DES2, DES3, DES4, DES5, and DES6 are 3.12 × 10^−10^, 2.98 × 10^−10^, 2.03 × 10^−10^, 1.91 × 10^−10^, 1.87 × 10^−10^, and 1.83 × 10^−10^ m^2^ s^−1^ at 303.15 K. However, at high temperatures (313.15 K), the diffusion coefficients are 5.12 × 10^−10^, 4.88 × 10^−10^, 3.62 × 10^−10^, 3.41 × 10^−10^, 3.25 × 10^−10^, and 3.08 × 10^−10^ m^2^ s^−1^. These values are comparable to the experimental values of 5.07 × 10^−10^ for DES1, 3.37 × 10^−10^ for DES4, and 3.01 × 10^−10^ for DES6 at 313.15 K.^61^Fig. 10 shows how raising the absorption temperature considerably reduces CO_2_ solubility while increasing the diffusion rate. The diffusivities of CO_2_ in DES1 and DES2 were found to be higher than those of other DESs, which is consistent with values reported in the literature.^61^ This result implies that crucial factors in developing CO_2_ absorption based on DESs are the DES molecular structure and operating conditions.

### 3.5. COSMO-RS prediction

**Fig. 11:**
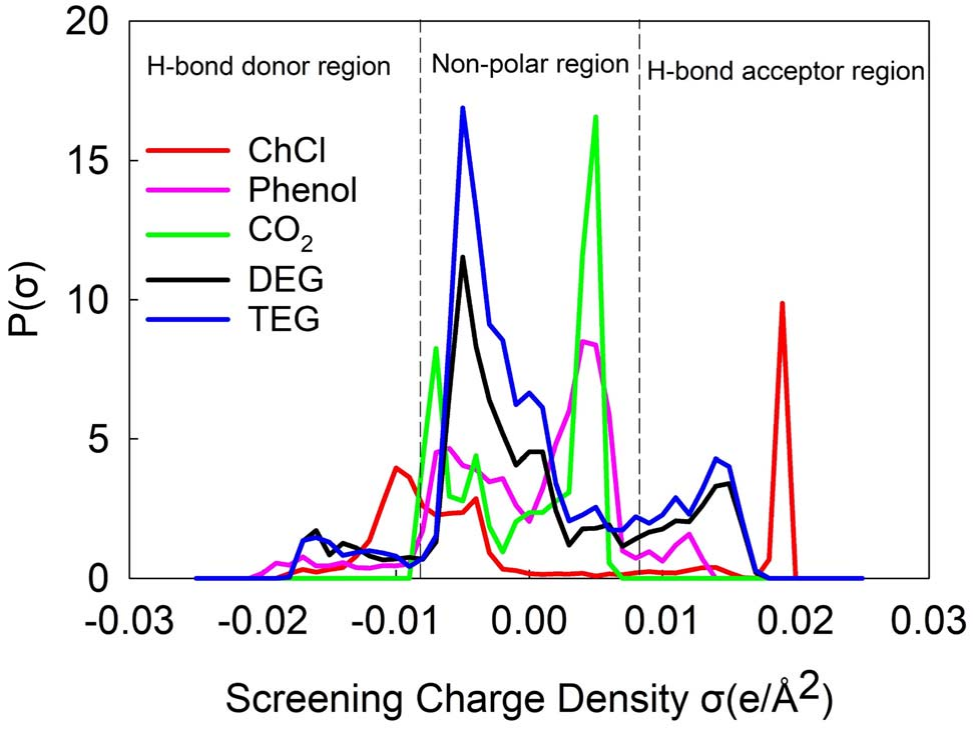
Sigma profiles of HBDs, HBAs, and CO_2_.

The strength of molecular interactions between two compounds is determined by their complementary σ-profiles. When one compound's σ-profile increases while the other's decreases in the same region, the interactions become stronger. In this work, the HBDs of phenol, DEG, and TEG, as well as the HBAs of ChCl, were chosen to predict the σ-profiles in order to investigate the impacts of HBAs and HBDs on the interactions with CO_2_. Fig. 11 shows the predicted results. According to Liu *et al.*,^62^ the σ-profiles may be separated into three regions: the H-bond acceptor region (σ > +0.0082 e Å^−2^), the non-polar region (−0.0082 < σ < +0.0082 e Å^−2^), and the H-bond donor region (σ < −0.0082 e Å^−2^). ChCl has a high interaction with CO_2_, as evidenced by the broader region in its σ-profile that is augmented by CO_2_ in both non-polar and H-bond donor regions. In the meantime, the interaction strength with CO_2_ is also reflected in the position of the σ-profile peak in the H-bond acceptor area. Furthermore, as illustrated in Fig. 11, the σ-profile of HBD for TEG has a larger region complemented with CO_2_ than that of DEG and phenol in both non-polar and H-bond acceptor regions. A further indication of the intensity of the interaction with CO_2_ is the position of the σ-profile peak in the H-bond donor and H-bond acceptor area. As demonstrated by the σ-profile prediction, the CO_2_ solubility can be influenced by the various HBDs. This is consistent with the experimental findings^43^ of *X*_CO_2__(ChCl–TEG 1 : 4) > *X*_CO_2__(ChCl–TEG 1 : 3) > *X*_CO_2__(ChCl–phenol 1 : 3) and *X*_CO_2__(ChCl–phenol 1 : 4) > *X*_CO_2__(ChCl–DEG 1 : 4) > *X*_CO_2__(ChCl–DEG 1 : 3) at the same temperature and pressure. Therefore, the σ-profile can be used to represent how strongly a DES interacts with CO_2_.

The Henry's constants of CO_2_ in DESs were computed using the COSMO-RS model and compared with the experimental results.^43^Table 7 lists the precise values of the expected and experimental Henry's constants. It is evident from Table 7 that the expected Henry's constants rise with temperature and match the experimental findings. Most of the predicted Henry's constants are lower than the observed values because Henry's constant is inversely proportional to CO_2_ solubility. This is consistent with the CO_2_ solubility observation. When solubility and Henry's constants are compared, the solubility of CO_2_ is greatest in DESs of triethylene glycol and choline chloride with a mole ratio of 1 : 4, but the values of Henry's constants are lowest at the same temperature.

**Table 7:** Comparing the Henry's law constant of CO_2_ (MPa) at various temperatures with the available literature^43^

Systems	*H*_x_ (MPa)		
	*T* = 303 K	*T* = 313 K	*T* = 323 K
DES1	23.7 (29.9)	25.5 (33.1)	32.1 (39.3)
DES2	25.7 (31.1)	27.8 (33.0)	33.3 (40.1)
DES3	28.3 (30.9)	31.8 (36.4)	38.8 (45.6)
DES4	27.8 (31.5)	30.2 (35.4)	36.5 (44.5)
DES5	17.4 (22.5)	22.3 (27.1)	28.4 (32.3)
DES6	13.7 (20.9)	18.7 (25.5)	24.7 (31.8)

## 4. Conclusions

This work presents a comprehensive multi-scale computational analysis that uses MD simulations and the COSMO-RS model to investigate the mechanism and efficiency of CO_2_ capture in different ChCl-based DESs. We developed a direct correlation between nanoscale structural arrangements, interfacial behavior, and bulk thermodynamic properties by systematically varying the hydrogen bond donor type (phenol, DEG, and TEG) and HBA : HBD molar ratios (1 : 3 and 1 : 4), and our findings were in excellent agreement with available experimental data. Interfacial analysis using MD simulations, density profiles revealed that CO_2_ preferentially accumulates at the gas–liquid interface before being physically dissolved into the bulk phase. Bulk phase solubility trends show that the CO_2_ capture capacity is highly influenced by the chemical structure of the HBD, decreasing with increasing temperature—a property consistent with a physical dissolution process. Among all studied systems, the DES6 system (ChCl : TEG at a 1 : 4 ratio) had the highest bulk CO_2_ density and overall solubility, a macroscopic performance supported by both the microstructural and phase-equilibrium insights obtained in this study. At the molecular level, a clear integration of structural and energetic properties explains why TEG-based systems have a higher affinity for the gas. RDFs and SDFs reveal that the pure ChCl–TEG system has weaker interspecies interactions, resulting in bigger intrinsic interstitial cavities that can easily accommodate CO_2_ molecules. Structural research reveals that the dissolved CO_2_ carbon atom is surrounded by a circular shell of Cl^−^ anions and HBD molecules, whereas [Ch]^+^ cations coordinate symmetrically with the CO_2_ oxygen atoms. These results indicate that CO_2_–HBD interactions dominate ion–CO_2_ interactions in all systems, with DES6 having the highest favorable interaction energy and the densest network of physical hydrogen bonds.

The COSMO-RS thermodynamic model offers an electronic-level explanation for the observed solubility limits in addition to these explicit MD findings. The projected σ-profiles show that the TEG HBD has a significantly broader and more electrically complementary landscape than CO_2_ in both non-polar and H-bond acceptor areas when compared to phenol and DEG. This electrical complementarity corresponds to the predicted and observed solubility trend of DES6 > DES5 > DES1 > DES2 > DES4 > DES3. Furthermore, the predicted Henry's law constants quantitatively correspond to these solubility limits, with DES6 consistently producing the lowest Henry's constants across all simulated temperatures. Transport properties measured using MSD demonstrate a classic trade-off within these structural frameworks, as higher operating temperatures accelerate CO_2_ diffusion coefficients while severely penalizing the overall thermodynamic solubility limit. Our integrated multi-scale method effectively correlates electronic-structure properties (σ-profiles) and molecular topologies (RDFs and SDFs) with bulk transport and thermodynamic parameters, including Henry's constants, diffusivity, and solubility. The remarkable correlation between the local structural mechanics described by MD and the phase-equilibrium thermodynamics predicted by COSMO-RS confirms this computational framework as a reliable computational tool. This methodology provides an effective predictive workflow for screening and optimizing deep eutectic solvents for green gas separation technologies, promoting more theoretical research into high-performance carbon capture medium.

## Author contributions

Prateek Banerjee was involved in writing – original draft, methodology, investigation, formal analysis, data collection, visualization, and conceptualization. Surya Siddaraje Urs was involved in methodology, investigation, formal analysis, data collection, conceptualization. Rima Biswas was involved in writing – review and editing, supervision, validation, investigation, formal analysis, data collection, visualization, methodology, and conceptualization.

## Conflicts of interest

There are no conflicts to declare.

## Data Availability

The datasets used and/or analysed during the current study available from the corresponding author on reasonable request.

## References

[cit1] Gür T. M. (2022). Carbon Dioxide Emissions, Capture, Storage and Utilization: Review of Materials, Processes and Technologies. Prog. Energy Combust. Sci..

[cit2] Gao W., Liang S., Wang R., Jiang Q., Zhang Y., Zheng Q., Xie B., Toe C. Y., Zhu X., Wang J., Huang L., Gao Y., Wang Z., Jo C., Wang Q., Wang L., Liu Y., Louis B., Scott J., Roger A.-C., Amal R., He H., Park S.-E. (2020). Industrial carbon dioxide capture and utilization: state of the art and future challenges. Chem. Soc. Rev..

[cit3] Aghel B., Janati S., Wongwises S., Shadloo M. S. (2022). Review on CO2 capture by blended amine solutions. Int. J. Greenhouse Gas Control.

[cit4] Meng F., Meng Y., Ju T., Han S., Lin L., Jiang J. (2022). Research progress of aqueous amine solution for CO2 capture: A review. Renew. Sustain. Energy Rev..

[cit5] Taib M. M., Murugesan T. (2012). Solubilities of CO2 in aqueous solutions of ionic liquids (ILs) and monoethanolamine (MEA) at pressures from 100 to 1600kPa. Chem. Eng. J..

[cit6] Damanafshan M., Mokhtarani B., Mirzaei M., Sharifi A. (2021). Equilibrium solubility measurement of carbon dioxide in hybrid solvents of aqueous N-methyldiethanolamine blended with 1-Methyl-3-octyl-imidazolium tetrafluoroborate ionic liquid at high pressures. J. Mol. Liq..

[cit7] Shama V. M., Swami A. R., Aniruddha R., Sreedhar I., Reddy B. M. (2021). Process and engineering aspects of carbon capture by ionic liquids. J. CO_2_ Util..

[cit8] Noorani N., Mehrdad A., Ahadzadeh I. (2021). CO2 absorption in amino acid-based ionic liquids: Experimental and theoretical studies. Fluid Phase Equilib..

[cit9] Kang S., Chung Y. G., Kang J. H., Song H. (2020). CO2 absorption characteristics of amino group functionalized imidazolium-based amino acid ionic liquids. J. Mol. Liq..

[cit10] Song Z., Hu X., Zhou Y., Zhou T., Qi Z., Sundmacher K. (2019). Rational design of double salt ionic liquids as extraction solvents: Separation of thiophene/n-octane as example. AIChE J..

[cit11] Zhao Y., Dong Y., Guo Y., Huo F., Yan F., He H. (2021). Recent progress of green sorbents-based technologies for low concentration CO2 capture. Chin. J. Chem. Eng..

[cit12] Aghaie M., Rezaei N., Zendehboudi S. (2018). A systematic review on CO2 capture with ionic liquids: Current status and future prospects. Renew. Sustain. Energy Rev..

[cit13] Song Z., Hu X., Wu H., Mei M., Linke S., Zhou T., Qi Z., Sundmacher K. (2020). Systematic Screening of Deep Eutectic Solvents as Sustainable Separation Media Exemplified by the CO2 Capture Process. ACS Sustainable Chem. Eng..

[cit14] Leron R. B., Li M.-H. (2012). High-pressure density measurements for choline chloride: Urea deep eutectic solvent and its aqueous mixtures at T=(298.15 to 323.15)K and up to 50MPa. J. Chem. Thermodyn..

[cit15] El Achkar T., Greige-Gerges H., Fourmentin S. (2021). Basics and properties of deep eutectic solvents: a review. Environ. Chem. Lett..

[cit16] Abbott A. P., Capper G., Davies D. L., Rasheed R. K., Tambyrajah V. (2003). Novel solvent properties of choline chloride/urea mixtures. Commun. Chem..

[cit17] Shah P. A., Chavda V., Hirpara D., Sharma V. S., Shrivastav P. S., Kumar S. (2023). Exploring the potential of deep eutectic solvents in pharmaceuticals: Challenges and opportunities. J. Mol. Liq..

[cit18] Francisco M., van den Bruinhorst A., Kroon M. C. (2012). New natural and renewable low transition temperature mixtures (LTTMs): screening as solvents for lignocellulosic biomass processing. Green Chem..

[cit19] Smith E. L., Abbott A. P., Ryder K. S. (2014). Deep Eutectic Solvents (DESs) and Their Applications. Chem. Rev..

[cit20] Leron R. B., Li M.-H. (2013). Solubility of carbon dioxide in a choline chloride–ethylene glycol based deep eutectic solvent. Thermochim. Acta.

[cit21] Bhawna, Pandey A., Pandey S. (2017). Superbase-Added Choline Chloride-Based Deep Eutectic Solvents for CO2 Capture and Sequestration. ChemistrySelect.

[cit22] Chen Y., Han X., Liu Z., Yu D., Guo W., Mu T. (2020). Capture of Toxic Gases by Deep Eutectic Solvents. ACS Sustainable Chem. Eng..

[cit23] Li X., Hou M., Han B., Wang X., Zou L. (2008). Solubility of CO2 in a Choline Chloride + Urea Eutectic Mixture. J. Chem. Eng. Data.

[cit24] Francisco M., van den Bruinhorst A., Zubeir L. F., Peters C. J., Kroon M. C. (2013). A new low transition temperature mixture (LTTM) formed by choline chloride+lactic acid: Characterization as solvent for CO2 capture. Fluid Phase Equilib..

[cit25] Chen Y., Ai N., Li G., Shan H., Cui Y., Deng D. (2014). Solubilities of Carbon Dioxide in Eutectic Mixtures of Choline Chloride and Dihydric Alcohols. J. Chem. Eng. Data.

[cit26] Leron R. B., Caparanga A., Li M.-H. (2013). Carbon dioxide solubility in a deep eutectic solvent based on choline chloride and urea at T=303.15–343.15K and moderate pressures. J. Taiwan Inst. Chem. Eng..

[cit27] Jiang C., Cheng H., Qin Z., Wang R., Chen L., Yang C., Qi Z., Liu X. (2021). COSMO-RS prediction and experimental verification of 1,5-pentanediamine extraction from aqueous solution by ionic liquids. Green Energy Environ..

[cit28] Haghbakhsh R., Raeissi S. (2018). Modeling vapor-liquid equilibria of mixtures of SO2 and deep eutectic solvents using the CPA-NRTL and CPA-UNIQUAC models. J. Mol. Liq..

[cit29] Crespo E. A., Silva L. P., Lloret J. O., Carvalho P. J., Vega L. F., Llovell F., Coutinho J. A. P. (2019). A methodology to parameterize SAFT-type equations of state for solid precursors of deep eutectic solvents: the example of cholinium chloride. Phys. Chem. Chem. Phys..

[cit30] Haider M. B., Kumar R. (2020). Solubility of CO2 and CH4 in sterically hindered amine-based deep eutectic solvents. Sep. Purif. Technol..

[cit31] Han J., Dai C., Yu G., Lei Z. (2018). Parameterization of COSMO-RS model for ionic liquids. Green Energy Environ..

[cit32] Yang J., Hou Z., Wen G., Cui P., Wang Y., Gao J. (2019). A Brief Review of the Prediction of Liquid–Liquid Equilibrium of Ternary Systems Containing Ionic Liquids by the COSMO-SAC Model. J. Solution Chem..

[cit33] Liu Y., Yu H., Sun Y., Zeng S., Zhang X., Nie Y., Zhang S., Ji X. (2020). Screening Deep Eutectic Solvents for CO2 Capture With COSMO-RS. Front. Chem..

[cit34] Pour S. B., Dabbagh Hosseini pour M., Jahanbin Sardroodi J., Rastkar Ebrahimzadeh A., Pazuki G., Avestan M. S. (2024). Exploring the efficiency of caprylic acid and quaternary ammonium salt-based deep eutectic solvents for separation of CO2 and H2S from gas mixtures using molecular dynamics simulations and COSMO-RS. Chem. Eng. J. Adv..

[cit35] Kamgar A., Mohsenpour S., Esmaeilzadeh F. (2017). Solubility prediction of CO2, CH4, H2, CO and N2 in Choline Chloride/Urea as a eutectic solvent using NRTL and COSMO-RS models. J. Mol. Liq..

[cit36] Salehi H. S., Hens R., Moultos O. A., Vlugt T. J. H. (2020). Computation of gas solubilities in choline chloride urea and choline chloride ethylene glycol deep eutectic solvents using Monte Carlo simulations. J. Mol. Liq..

[cit37] Hens R., Vlugt T. J. H. (2018). Molecular Simulation of Vapor–Liquid Equilibria Using the Wolf Method for Electrostatic Interactions. J. Chem. Eng. Data.

[cit38] Ullah R., Atilhan M., Anaya B., Khraisheh M., García G., ElKhattat A., Tariq M., Aparicio S. (2015). A detailed study of cholinium chloride and levulinic acid deep eutectic solvent system for CO2 capture via experimental and molecular simulation approaches. Phys. Chem. Chem. Phys..

[cit39] García G., Atilhan M., Aparicio S. (2015). Interfacial Properties of Deep Eutectic Solvents Regarding to CO2 Capture. J. Phys. Chem. C.

[cit40] Altamash T., Atilhan M., Aliyan A., Ullah R., García G., Aparicio S. (2016). Insights into choline chloride–phenylacetic acid deep eutectic solvent for CO2 absorption. RSC Adv..

[cit41] Malik A., Dhattarwal H. S., Kashyap H. K. (2021). Distinct Solvation Structures of CO2 and SO2 in Reline and Ethaline Deep Eutectic Solvents Revealed by AIMD Simulations. J. Phys. Chem. B.

[cit42] Alizadeh V., Esser L., Kirchner B. (2021). How is CO2 absorbed into a deep eutectic solvent?. J. Phys. Chem..

[cit43] Li G., Deng D., Chen Y., Shan H., Ai N. (2014). Solubilities and thermodynamic properties of CO2 in choline-chloride based deep eutectic solvents. J. Chem. Thermodyn..

[cit44] Phillips J. C., Braun R., Wang W., Gumbart J., Tajkhorshid E., Villa E., Chipot C., Skeel R. D., Kalé L., Schulten K. (2005). Scalable molecular dynamics with NAMD. J. Comput. Chem..

[cit45] Li W., Xie H., Huang Y., Song L., Shao Y., Qiu K. (2016). Application of Gaussian 09/GaussView 5.0 in analytical chemistry teaching. J. Kunming Med. Univ..

[cit46] Martínez L., Andrade R., Birgin E. G., Martínez J. M. (2009). PACKMOL: A package for building initial configurations for molecular dynamics simulations. J. Comput. Chem..

[cit47] Potoff J. J., Siepmann J. I. (2001). Vapor–liquid equilibria of mixtures containing alkanes, carbon dioxide, and nitrogen. AIChE J..

[cit48] Doherty B., Acevedo O. (2018). OPLS Force Field for Choline Chloride-Based Deep Eutectic Solvents. J. Phys. Chem. B.

[cit49] Humphrey W., Dalke A., Schulten K. (1996). VMD: Visual molecular dynamics. J. Mol. Graphics.

[cit50] Brehm M., Kirchner B. (2011). TRAVIS - A Free Analyzer and Visualizer for Monte Carlo and Molecular Dynamics Trajectories. J. Chem. Inf. Model..

[cit51] Heydari Dokoohaki M., Zolghadr A. R. (2021). Significant Improvement in CO2 Absorption by Deep Eutectic Solvents as Immobilized Sorbents: Computational Analysis. J. Phys. Chem. B.

[cit52] Hess B., Kutzner C., van der Spoel D., Lindahl E. (2008). GROMACS
4: Algorithms for Highly Efficient, Load-Balanced, and Scalable Molecular Simulation. J. Chem. Theory Comput..

[cit53] Biswas R., Ghosh P., Banerjee T., Ali S. M. (2018). Partitioning of Cs+ and Na+ ions by dibenzo-18-crown-6 ionophore in biphasic aqueous systems of octanol and ionic liquid. Radiochim. Acta.

[cit54] Sarangi S. S., Zhao W., Müller-Plathe F., Balasubramanian S. (2010). Correlation between Dynamic Heterogeneity and Local Structure in a Room-Temperature Ionic Liquid: A Molecular Dynamics Study of [bmim][PF6]. ChemPhysChem.

[cit55] Klamt A. (1995). Conductor-like Screening Model for Real Solvents: A New Approach to the Quantitative Calculation of Solvation Phenomena. J. Phys. Chem..

[cit56] Klamt A., Schüürmann G. (1993). COSMO: a new approach to dielectric screening in solvents with explicit expressions for the screening energy and its gradient. J. Chem. Soc., Perkin Trans. 2.

[cit57] SADF , Theoretical Chemistry, Vrije Universiteit, Amsterdam, The Netherlands. 2012

[cit58] te Velde G., Bickelhaupt F. M., Baerends E. J., Fonseca Guerra C., van Gisbergen S. J. A., Snijders J. G., Ziegler T. (2001). Chemistry with ADF. J. Comput. Chem..

[cit59] Sumon K. Z., Henni A. (2011). Ionic liquids for CO2 capture using COSMO-RS: Effect of structure, properties and molecular interactions on solubility and selectivity. Fluid Phase Equilib..

[cit60] Roscioli J. R., Nesbitt D. J. (2010). State-Resolved Scattering at Room-Temperature Ionic Liquid−Vacuum Interfaces: Anion Dependence and the Role of Dynamic versus Equilibrium Effects. J. Phys. Chem. Lett..

[cit61] Xin K., van Sint Annaland M. (2023). Diffusivities and solubilities of carbon dioxide in deep eutectic solvents. Sep. Purif. Technol..

[cit62] Liu Y., Meyer A. S., Nie Y., Zhang S., Zhao Y., Fosbøl P. L., Thomsen K. (2017). Freezing Point Determination of Water–Ionic Liquid Mixtures. J. Chem. Eng. Data.

